# Renin-Angiotensin System Inhibitor Usage and Age-Related Macular Degeneration among Hypertensive Patients: Results from the National Health and Nutrition Examination Survey, 2005–2008

**DOI:** 10.1155/2020/4252031

**Published:** 2020-06-24

**Authors:** Chi Ren, Weiming Liu, Xue Yin, Bingyu Zhang, Peirong Lu

**Affiliations:** Department of Ophthalmology, The First Affiliated Hospital of Soochow University, 188 Shizi Street, Suzhou 215006, China

## Abstract

**Purpose:**

To assess whether renin-angiotensin system inhibitor (RASI) utilization is associated with age-related macular degeneration (AMD) prevalence among hypertensive patients.

**Methods:**

A US population-based, cross-sectional study was conducted. 3,023 hypertensive participants aged 40 years and older with gradable retinal images and ascertained RASI usage in the National Health and Nutrition Examination Survey (NHANES), 2005–2008, were finally enrolled into the study. RASI usage was obtained by interview, and AMD was determined through retinal image assessment. We performed multivariable analyses to assess the relationship between utilization of RASIs and AMD prevalence. We also took drug treatment duration into account, in order to better understand the effects of RASIs.

**Results:**

Multivariable logistic regression analyses revealed that AMD prevalence had no significant association with RASI usage but was inversely correlated with RASI treatment duration (odds ratio (OR) = 0.87, 95% confidence interval (CI) = 0.78–0.98, *p*=0.02). Long-term usage (>5 years) of RASIs was significantly associated with not only reduced overall risk of AMD (OR = 0.23, 95% CI = 0.14–0.38, *p* < 0.001) but also lower propensity to have early (OR = 0.23, 95% CI = 0.14–0.37, *p* < 0.001) and late (OR = 0.25, 95% CI = 0.07–0.87, *p*=0.03) AMD. Furthermore, long-term RASI users were less prone to develop soft drusen (OR = 0.67, 95% CI = 0.45–0.99, *p*=0.04) and geographic atrophy (GA) (OR = 0.39, 95% CI = 0.22–0.71, *p*=0.003).

**Conclusions:**

Evidence supporting that RASI utilization could directly protect against AMD in hypertensive patients was still insufficient, but long-term RASI treatment seemed to be beneficial for both early and late AMD, implicating a promising therapeutic approach that RASIs might offer for AMD prevention and management.

## 1. Introduction

Age-related macular degeneration (AMD) is a major cause of visual impairment and blindness among elderly people in developed countries [1], accounting for more than 50% of blindness in white population aged 40 years and older in the US [2]. Early AMD is characterized by drusen presence and pigmentary abnormalities, and late AMD has two subtypes, namely, geographic atrophy (GA) (“dry”) and choroidal neovascularization (CNV) (“wet”). It is well known that odds of AMD increase sharply as age increases, and with prolonged average lifespan expectancy, the prevalence of visual disability caused by AMD is projected to increase drastically [1, 3, 4], appealing for plenty of attention in the literature.

The mechanisms of AMD are not completely established yet. Besides age [1, 3, 5], AMD is reported to be related to ethnicity [5], cigarette consuming [6], lower levels of antioxidants [7], less physical activity [8], systemic inflammation [9], genetic factors [10, 11], etc. Apart from that, hypertension is also involved as a risk factor for AMD [5, 12], multiplying the difficulties in developing efficient AMD treatment approaches for hypertensive patients. Although a variety of agents are now approved for AMD (mainly for wet AMD) treatment, their therapeutic effects are limited and vision loss prevention is still challenging [13].

Renin-angiotensin system (RAS) plays vital roles in hypertension. Angiotensin II (Ang II), a crucial member of RAS, is also present in eye [14, 15] and is reported to promote AMD development through various mechanisms, such as inducing inflammation, oxidative stress, and endothelial dysfunctions [12, 16]. Two main categories of RAS inhibitors (RASIs) are currently in use, angiotensin-converting enzyme inhibitors (ACEIs) and Ang II receptor blockers (ARBs). The former targets angiotensin-converting enzyme (ACE), the key catalyst for the conversion of Ang I to Ang II, and the latter selectively blocks the binding of Ang II to one of its receptors, Ang II type 1 receptor (AT_1_R), to interrupt the biological function of Ang II. In some animal studies, RASIs were found to protect retina under pathological conditions, such as inflammation and diabetic status [17–19]. This evidence suggested a promising therapeutic approach that RASIs could offer for AMD treatment. However, till now, no benefits of RASI utilization in AMD patients have been proved by human trials [12, 20, 21], and there was even a report implicating deleterious impacts of RASIs [22]. Since RASIs are now the first-line choice for hypertension treatment, understanding the relationship between RASIs and AMD will undoubtedly facilitate AMD prevention and management, especially in those suffering from hypertension. Thus, further investigations are required to focus on the controversial issue.

Hence, we conducted a cross-sectional study to find out whether RASI utilization patterns are related to AMD prevalence among hypertensive individuals ≥40 years old using the data from the National Health and Nutrition Examination Survey (NHANES), 2005–2008.

## 2. Subjects and Methods

### 2.1. Data Source

We used data from the NHANES, an annual program conducted by Centres for Disease Control and Prevention's (CDC's) National Centre for Health Statistics (NCHS), which is designed to assess the health and nutritional status of the US noninstitutionalized population who were selected by a stratified multistage sampling design, with applications of house screeners, interviews, and physical and laboratory examinations. All the NHANES protocols complied with the Health and Human Services (HHS) Policy for Protection of Human Research Subjects [23] and were annually reviewed and approved by NCHS Research Ethics Review Board. All the survey participants gave their informed consent. Details about the NHANES study design and operations have been fully elaborated elsewhere [24], and other additional information about the survey is all available at the NHANES website [25].

### 2.2. Study Population

**Figure 1:**
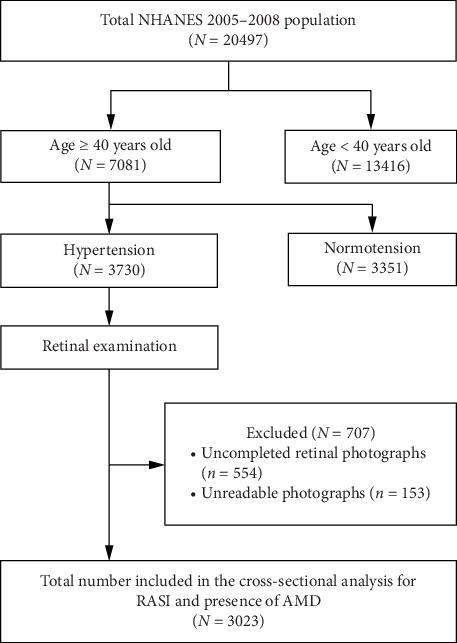
Population selection to obtain the final participants for assessment of the relationship between RASIs and AMD presence. NHANES: the National Health and Nutrition Examination Survey; RASI: renin-angiotensin system inhibitor; AMD: age-related macular degeneration.

Data from two NHANES cycles, 2005-2006 and 2007-2008, were used for the present cross-sectional study. Since RASIs are mostly used for hypertension treatment, we only enrolled hypertensive participants who were 40 years or older. Systemic hypertension would be identified when average systolic blood pressure ≥140 mmHg, average diastolic blood pressure ≥90 mmHg, or the question “have you ever been told by a doctor or other health professional that you had hypertension, also called high blood pressure?” in the self-reported questionnaire was answered with “Yes.” Identification of study population is demonstrated in Figure 1. In brief, of the total 20,497 participants, 7,081 participants aged ≥40 years went through fundus examination, among whom 3,730 had hypertension. 707 participants were further excluded and 3,023 were finally included in the cross-sectional study.

### 2.3. Retinal Imaging and Grading of Age-Related Macular Degeneration

A complete description of retinal photography and AMD grading system can be found elsewhere [26–28]. Briefly, the room was darkened for pupil dilation, and two 45-degree digital R6 nonmydriatic cameras (Canon USA, Inc., One Canon Park, Melville, New York) with a Canon EOS 10D camera back were used for fundus photography. Each image was assessed twice using the modified Wisconsin Age-Related Maculopathy Grading System [29]. AMD was classified into no AMD, early AMD, or late AMD according to the retinal image assessment, and the three severity levels were determined based on certain criteria: (1) no AMD was defined as gradable images with no manifestation relevant to AMD; (2) early AMD was defined as the presence of soft drusen with a greater area of 500 *μ*m or in the central circle and/or pigmentary abnormalities; (3) late AMD was defined as the presence of any late lesions, such as GA, exudative evidence related to AMD, or laser and/or photodynamic therapy for AMD. Data from the eye with worse disease manifestation would be used if data of both eyes were available.

### 2.4. Main Variable

Our main variable for this study was the self-reported RASI usage. Information about prescription medication of the participants was collected through interviews. Participants were asked whether they had taken any prescription medication in the past month. If the answer was “yes,” then they were further asked about the drug name, the usage duration, and the major reason for drug utilization. The drug names were obtained from the medication container label or verbal report, recorded by the interviewer and matched using the Lexicon Plus drug classification database (Cerner Multum, Inc., Kansas City, Missouri). Two categories of RASIs were ACEIs and ARBs. ACEIs identified in this study included captopril, enalapril, cilazapril, fosinopril, lisinopril, perindopril, ramipril, benazepril, moexipril, quinapril, and trandolapril, and ARBs included candesartan, eprosartan, irbesartan, losartan, telmisartan, valsartan, olmesartan, and azilsartan. Long-term usage was defined as taking RASIs for more than 5 years.

### 2.5. Other Variables

Data on demographic variables (age, gender, race, education level), health-related behaviors (cigarettes and alcohol consuming), general health condition, medical comorbidities (history of stroke, heart diseases, cancer or malignancy, arthritis, liver problem, thyroid problem), and ocular comorbidities (history of cataract extraction) were collected using self-reported questionnaire. Participants who reported any history of coronary heart disease, angina pectoris, myocardial infarction, or congestive heart failure would be determined as having heart disease history. Body mass index (BMI) was calculated from height and weight measured during physical examination at the time of survey. A subject would be identified as diabetes mellitus if he/she met any of the following criteria: (1) fasting plasma glucose ≥126 mg/ml (7.0 mmol/L), (2) 2-hour plasma glucose ≥200 mg/dl (11.1 mmol/L), (3) HbA1c ≥6.5%, (4) the answer to the question “have you ever been told by a doctor or health professional that you have diabetes or sugar diabetes?” being “Yes.” Data on total cholesterol, low- and high-density lipoprotein, and apolipoprotein B (ApoB) of each participant were obtained through laboratory examination of blood specimens.

### 2.6. Statistical Analyses

Data analyses were all conducted using STATA software (version 12.0, StataCorp LP, College Station, TX, USA) with sampling weigh following the survey analytic guideline to account for the stratified, complex sample design of the NHANES [30]. We used Taylor linearization to estimate the sample distribution and laboratory test results in each group of the US national population. To examine the relationship between RASI utilization patterns and prevalence of AMD, odds ratios (ORs) with their corresponding 95% confidence intervals (CIs) were calculated by survey-weighted univariable logistic regression analyses, and those variates which did not reach the significance of mean or distribution estimates would be excluded from the multivariable logistic regression model. Interactions between the confounding factors, e.g., age and systemic hypertension, were not taken into consideration in the multivariable regression analyses. We did not take subjects who took both ACEI and ARB into account when analyzing effects of different RASI categories, not only because of their lack in number but also to avoid obscuring the single effect of one specific drug category. A value of *p* < 0.05 was determined as statistically significant for all the analyses.

## 3. Results

### 3.1. Characteristics of Study Population

3,023 hypertensive participants (total weighted sample of 55,020,856 participants) aged 40 years and older with gradable retinal images were finally included in our study.  Table 1 summarizes the demographic characteristics and confounding commodities of the population. Of this total, 49.4% participants were aged 60 years and older, 46.7% were male, 75.5% were non-Hispanic white, and 8.4% suffered from any AMD. Compared with those without AMD, subjects identified with AMD were older and tended to have history of cataract surgery, heart diseases, and stroke, and their peripheral blood contained higher level of high-density lipoprotein (HDL) cholesterol. Moreover, we found a significant difference in the drug usage and category of RASIs among subjects identified with no, early, and late AMD.

### 3.2. RASI Utilization and AMD Prevalence in Hypertensive Population

First, we assessed whether RASI utilization had any effects on AMD prevalence using the data of the whole hypertensive population. No significant correlation was identified in the assessment, not only in age-adjusted model (OR = 1.17, 95% CI = 0.88–1.57, *p*=0.27) but also in multivariate-adjusted model (OR = 1.32, 95% CI = 0.83–2.08, *p*=0.22) (Table 2). When RASIs were categorized into ACEIs and ARBs, there were still no significant results. These results indicated that AMD might not be independently related to RASI intake.

The NHANES study classified the retinal images into no, early, and late AMD. Our results of multivariable analysis showed that RASI utilization had no remarkable influence on both early (*p*=0.33) and late (*p*=0.27) AMD, and the results remained insignificant when RASIs were categorized into ACEIs and ARBs (Table 3).

### 3.3. Duration of RASI Utilization and AMD Prevalence among RASI Users

Since the previous assessments only included the data obtained at one time point, without considering the potential cumulative effects of time, we next investigated whether different RASI treatment duration would make any difference among RASI users, but we did not test the long-term effect according to the type of RASI because of limited sample size. As shown in Table 4, odds for any AMD slightly decreased as RASI treatment duration elongated per year (OR = 0.87, 95% CI = 0.78–0.98, *p*=0.02). There was a trend toward a beneficial effect with lower OR for early AMD (OR = 0.87, 95% CI = 0.73–0.99, *p*=0.03), but not for late AMD (OR = 0.87, 95% CI = 0.73–1.05, *p*=0.14). The protective effect of RASIs was stronger in long-term users (>5 years of RASI utilization), not only on overall AMD (OR = 0.23, 95% CI = 0.14–0.38, *p* < 0.001), but also on early (OR = 0.23, 95% CI = 0.14–0.37, *p* < 0.001) and late (OR = 0.25, 95% CI = 0.07–0.87, *p*=0.03) AMD.

The results above suggested that long-term RASI usage might exert greater influence, so we finally focused on its effects on AMD subtypes (Table 5). For early AMD, we took two major manifestations, pigmentary abnormalities and soft drusen, into consideration. The multivariable analysis revealed a lower possibility of long-term RASI users to develop soft drusen (OR = 0.67, 95% CI = 0.45–0.99, *p*=0.04), rather than pigmentary abnormalities (*p*=0.21). For late AMD, we identified a remarkably reduced odds for GA development among long-term RASI users (OR = 0.39, 95% CI = 0.22–0.71, *p*=0.003), but no significance for exudative AMD (*p*=0.73).

## 4. Discussion

Based on the data from the NHANES, 2005–2008, although there was insufficient evidence for determining the association between RASI usage and AMD prevalence, we found a less tendency of long-term RASI users to develop both early and late AMD, especially soft drusen and GA. Our results revealed a potential application prospect of RASIs in AMD prevention and treatment for hypertensive patients.

Several experimental studies have reported inhibited AMD development and progression by RASIs and suggested that RASIs become useful prophylaxes against CNV. Nagai et al. [31] demonstrated suppressed CNV by AT_1_R blockade in human samples and the murine model of laser-induced CNV, indicating an anti-inflammatory role of telmisartan via restraint of macrophage infiltration and downregulation of inflammatory cytokines such as vascular endothelial growth factor (VEGF), intercellular adhesion molecule-1 (ICAM-1), monocyte chemoattractant protein-1 (MCP-1), and interleukin-6 (IL-6) in the retinal pigment epithelium- (RPE-) choroid complex. Pons et al. [32] found the downregulation of matrix metalloproteinase-2 (MMP-2) and its key regulator, MMP-14, in Ang II pretreated mice and ARPE-19 cells by candesartan, providing a better understanding of the molecular events by which ARBs help delay the progression of sub-RPE deposits in AMD patients with hypertension. Li and Wang [33] identified a partial involvement of CD26/SDF-1 signaling pathway in the antiangiogenic effects of imidapril on laser-induced CNV. Although the use of ACEI might cause an increase in bradykinin, the effect of bradykinin on the of CNV development seemed to be minimal compared with the RAS [34].

However, till now, no population-based investigations have achieved positive results supporting the significant role of RASI usage in AMD prevention, including our present study. From our point of view, there may be several explanations for this discrepancy. To begin with, when assessing the relationship of RASI usage and AMD, we first only used data obtained at one time point about whether the participant took RASIs or not, without considering the accumulative effects of drug treatment time. Another important reason could be the differences in drug administration between animal experiments and human trials. Drug doses in animal experiments were often far greater than those in human trials. For example, in some animal studies, the individual doses of ARBs and ACEIs causing CNV regression were 0.5–10 mg/kg [31] and 1–10 mg/kg [34] per day, respectively, while in human trials, the daily dosage for a 70 kg-adult was only 0.035–0.3 mg/kg [21]. Besides, various ways of drug delivery would influence the results as well. Intraperitoneal or subcutaneous injection in animal models provided stronger pharmacokinetics and bioavailability of RASIs than oral administration in humans.

To our knowledge, the present study was the first to shed light on whether RASI treatment duration could make any difference in AMD prevention. Our analysis detected slightly lower possibility for the hypertensive patients to develop AMD as RASI medication time increases per year, and the OR for AMD was even lower among long-term users. These results were in line with the beneficial role of RASIs identified by experimental studies, but they further indicated a subtle but not immediate influence that normal dose of RASI treatment had on AMD management in hypertensive patients. Nevertheless, validation of this effect is still required in future studies.

Another finding of our study was that the risk of early AMD decreased with the time of RASI treatment. Furthermore, taking RASIs over 5 years seemed to exert remarkably protective effects against both early and late AMD, and the OR for early AMD was lower than that for late AMD. These results suggested a greater influence of RASIs on early AMD than late AMD. The two major manifestations of early AMD include soft drusen and pigmentary abnormalities, while benefits of RASIs were seen much more extended on soft drusen suppression. Soft drusen are accumulations of extracellular material immediately beneath the RPE layer, mainly containing ApoE, vitronectin, complement factor H (CFH), unesterified and esterified cholesterol, oil red O-bindings, and hydroxyapatite [35]. Lipoprotein deposition is one of the major mechanisms of drusen biogenesis [36]. Plasma lipoproteins and cholesterol from outer segments of photoreceptors are considered as important sources of lipoproteins making up soft drusen [37]. Borghi et al. [38] discussed the link between hypertension and hypercholesterolemia considering the metabolic pathways and the pathogenetic mechanisms connecting the two risk factors, supposing that RAS could be the critical mediator of this link, and this point also supported the rationale of RASIs inhibiting drusen formation in hypertension patients. Altered homeostasis of heavy metals like iron and zinc can also promote drusen formation [39–41]. It was reported that serum zinc concentration decreased following the use of ACEI in hypertensive patients [42], but the effects of RASIs on ocular heavy metal metabolism still need verification by deeper investigations.

It was of interest to note that our analysis revealed that long-term usage of RASIs had a greater protective association with GA rather than exudative AMD. This was understandable because drusen, which were found to be inhibited in long-term users, were strongly implicated as a causative factor of GA [35], yet little studies had verified this point. Since there is no effective treatment to prevent the onset and progression of GA currently, our results suggested a potential therapy for GA, and future studies should focus on the therapeutic effects and underlying mechanisms of RASIs protecting against GA.

It is worth mentioning that there seemed to be contradicted results as RASI usage showed a tendency to increase the risk of AMD (ORs >1 in Tables 2 and 3), but longer RASI usage was found to be beneficial (ORs <1 in Tables 4 and 5). Although ORs for RASI usage and AMD seemed to show bad effects, all of the results reached no statistical significance, so we could preliminary suppose that there were no significant effects. Besides, the reference groups were different: RASI nonusers in Tables 2 and 3, but short-term users in Tables 4 and 5, and our results highlighted the beneficial effects of long-term RASI treatment, so the results were not completely contradicted.

However, our study still had some limitations. First, the information provided by cross-sectional design was limited, as we had no way to know about those subjects who changed their medication, lifestyle, diet, or drug dose before the survey. Second, people who suffered from visual impairment due to AMD were less likely to take part in the complete survey, enhancing the selective bias in our study. Third, we excluded RASI nonusers when analyzing the relationship between RASI treatment duration and AMD prevalence, which might influence the analytic results to some extent due to a slight change of group distribution. Fourth, the interactions among different antihypertensive drugs were not taken into consideration in our analysis, so the effects of RASIs might be overvalued to some extent in patients receiving combination therapy. Finally, as hypertension is a critical risk factor of AMD, we cannot rule out the possibility that long-term usage of RASIs exerts protective effects indirectly via attenuating damage caused by hypertension instead of directly inhibiting AMD onset and progression.

## 5. Conclusion

Using data from the NHANES, 2005–2008, our study suggested that long-term RASI could be beneficial for both early and late AMD. Drusen formation and GA seemed more likely to be inhibited by long-term RASI treatment. Further investigations are required to clarify the mechanism of these correlations, in order to facilitate AMD prevention and management.

## Figures and Tables

**Table 1:** Characteristics of the study population.

Characteristics	AMD			*p*
	No AMD (*n* = 2,727)	Early AMD (*n* = 256)	Late AMD (*n* = 40)	
Demographics				
Age, years (%)				<0.001
40–54	40.9 (±1.7)	13.6 (±3.5)	1.9 (±1.9)	
55–64	26.8 (±1.1)	16.3 (±3.1)	5.3 (±3.5)	
65–74	20.2 (±0.9)	28.7 (±3.3)	5.7 (±4.0)	
75–84	11.4 (±0.9)	35.6 (±3.6)	74.9 (±9.0)	
≥85	0.7 (±0.1)	5.8 (±1.1)	12.2 (±5.9)	
Gender (%)				0.07
Male	46.7 (±1.1)	49.1 (±3.9)	24.9 (±7.7)	
Race (%)				0.25
Non-Hispanic white	75.5 (±2.4)	85.4 (±2.8)	93.5 (±3.4)	
Non-Hispanic black	12.6 (±1.8)	5.8 (±1.5)	2.4 (±2.0)	
Mexican American	4.3 (±0.7)	4.2 (±1.2)	0.9 (±0.9)	
Other races	7.6 (±0.9)	4.6 (±2.0)	3.2 (±2.5)	
Education level (%)				0.20
<9th grade	7.8 (±0.9)	12.2 (±3.7)	13.4 (±4.4)	
9–11th grade (including 12th grade with no diploma)	12.6 (±0.9)	17.1 (±3.7)	12.4 (±5.5)	
High school graduate/GED or equivalent	28.3 (±1.3)	26.7 (±3.5)	28.4 (±6.2)	
Some college or associate degree	27.0 (±1.3)	26.5 (±3.9)	29.9 (±6.4)	
College graduate or above	24.3 (±1.8)	17.4 (±3.0)	15.9 (±4.9)	
Health-related behaviors				
Consuming ≥100 cigarettes in life (%)	52.5 (±1.5)	55.8 (±3.2)	44.9 (±9.7)	0.48
Alcohol consumed in life ≥12 drinks (%)	17.6 (±1.1)	17.4 (±3.3)	3.1 (±3.1)	0.61
Systemic and ocular health conditions				
General health condition (%)				0.16
Excellent or good	76.4 (±1.4)	69.2 (±6.4)	100	
Fair	20.0 (±1.0)	24.0 (±4.6)	0	
Poor	3.6 (±0.6)	6.8 (±3.3)	0	
Body mass index (%)				0.12
<25	21.4 (±0.9)	22.0 (±2.6)	34.7 (±11.0)	
25–30	33.3 (±1.2)	36.0 (±3.0)	44.2 (±7.6)	
>30	45.3 (±1.3)	42.2 (±3.4)	21.1 (±8.2)	
Previous cataract surgery (%)	11.0 (±0.7)	31.1 (±3.6)	67.3 (±9.5)	<0.001
Diabetes mellitus (%)	23.0 (±1.2)	27.9 (±4.5)	29.3 (±6.3)	0.31
Heart diseases history (%)	14.5 (±1.3)	24.7 (±4.3)	33.2 (±12.7)	0.02
Stroke history (%)	6.0 (±0.7)	17.0 (±5.5)	22.6 (±14.6)	<0.01
Cancer or malignancy (%)	13.6 (±1.1)	16.6 (±4.3)	31.2 (±11.0)	0.13
Arthritis (%)	44.8 (±1.6)	50.0 (±5.8)	69.6 (±9.3)	0.13
Liver problem (%)	4.9 (±1.0)	3.8 (±1.9)	0	0.57
Thyroid problem (%)	16.0 (±1.7)	21.3 (±4.5)	27.2 (±10.6)	0.47
LDL cholesterol (mg/dL)	114.9 (±1.2)	109.7 (±3.4)	120.9 (±8.6)	0.27
Apolipoprotein (B) (mg/dL)	100.9 (±0.9)	96.5 (±2.62)	98.1 (±7.18)	0.24
Direct HDL cholesterol (mg/dL)	52.6 (±0.44)	55.3 (±0.99)	58.5 (±2.22)	<0.01
RASIs				
RASI utilization (%)	42.2 (±0.9)	49.3 (±3.7)	64.5 (±6.3)	<0.01
RASI category (%)^†^				0.01
ACEI	25.8 (±1.0)	32.8 (±3.1)	40.8 (±7.2)	
ARB	15.5 (±1.0)	15.9 (±2.7)	12.2 (±4.3)	

^†^Except participants taking both ACEI and ARB. AMD: age-related macular degeneration; GED: general equivalency diploma/general educational development; LDL: low-density lipoprotein; HDL: high-density lipoprotein; RASI: renin-angiotensin system inhibitor; ACEI: angiotensin-converting enzyme inhibitor; ARB: angiotensin receptor blocker; CI: confidence interval; SE: standard error. *Note.* Data are presented by weight-adjusted proportion (±SE) or mean (±SE).

**Table 2:** Age- and multivariate-adjusted ORs for any AMD among the study population.

	Age-adjusted		Multivariate-adjusted^‡^	
	OR (95% CI)	*p*	OR^†^ (95% CI)	*p*
RASI usage	1.17 (0.88–1.57)	0.27	1.32 (0.83–2.08)	0.22
RASI category^†^				
ACEI	1.31 (0.98–1.74)	0.06	1.37 (0.94–2.00)	0.09
ARB	0.93 (0.59–1.48)	0.76	1.22 (0.53–2.79)	0.62

^‡^Adjusted for age, stroke history, cataract surgery history, heart disease, and HDL. ^†^Except participants taking both ACEI and ARB. *Reference group*: RASI nonusers.. OR: odds ratio; AMD: age-related macular degeneration; CI: confidence interval; RASI: renin-angiotensin system inhibitor; ACEI: angiotensin-converting enzyme inhibitor; ARB: angiotensin receptor blocker; HDL: high-density lipoprotein.

**Table 3:** Multivariate-adjusted ORs for AMD among the hypertensive population.

	Early AMD		Late AMD	
	OR (95% CI)	*p*	OR (95% CI)	*p*
RASI usage	1.28 (0.76–2.15)	0.33	1.76 (0.62–5.02)	0.27
RASI category^†^				
ACEI	1.27 (0.80–2.05)	0.28	2.50 (0.86–7.30)	0.09
ARB	1.32 (0.57–3.01)	0.49	2.50 (0.86–7.30)	0.29

^†^Except participants taking both ACEI and ARB. Adjusted for age, stroke history, cataract surgery history, heart disease, and HDL. *Reference group*: RASI nonusers. OR: odds ratio; AMD: age-related macular degeneration; CI: confidence interval; RASI: renin-angiotensin system inhibitor; ACEI: angiotensin-converting enzyme inhibitor; ARB: angiotensin receptor blocker; HDL: high-density lipoprotein.

**Table 4:** Multivariate-adjusted ORs for any AMD in relation to RASI treatment duration among RASI users.

	Any AMD		Early AMD		Late AMD	
	OR (95% CI)	*p*	OR (95% CI)	*p*	OR (95% CI)	*p*
Per year	0.87 (0.78–0.98)	0.02	0.87 (0.77–0.99)	0.03	0.87 (0.73–1.05)	0.14
Long-term use^ǂ^	0.23 (0.14–0.38)	<0.001	0.23 (0.14–0.37)	<0.001	0.25 (0.07–0.87)	0.03

^ǂ^Long-term use was defined as taking RASI for more than 5 years. *Reference group:* patients taking RASIs for less than 5 years. Adjusted for age, stroke history, cataract surgery history, heart disease, and HDL. OR: odds ratio; AMD: age-related macular degeneration; CI: confidence interval; RASI: renin-angiotensin system inhibitor; HDL: high-density lipoprotein.

**Table 5:** Multivariate-adjusted ORs for AMD subtypes in relation to long-term RASI treatment among RASI users.

	OR (95% CI)	*p*
Early AMD manifestations		
Pigmentary abnormalities	0.76 (0.49–1.18)	0.21
Any soft drusen	0.67 (0.45–0.99)	0.04

Late AMD subtypes		
Exudative AMD (wet)	1.24 (0.35–4.36)	0.73
Geographic atrophy (dry)	0.39 (0.22–0.71)	0.003

Adjusted for age, stroke history, cataract surgery history, heart disease, and HDL. Long-term use was defined as taking RASI for more than 5 years. *Reference group:* patients taking RASIs for less than 5 years. OR: odds ratio; AMD: age-related macular degeneration; CI: confidence interval; RASI: renin-angiotensin system inhibitor; HDL: high-density lipoprotein.

## Data Availability

All the data used in the present study are available on https://wwwn.cdc.gov/nchs/nhanes/default.aspx.
